# Prevalence of the Antibiotic Resistance Genes in Coagulase-Positive-and Negative-*Staphylococcus* in Chicken Meat Retailed to Consumers

**DOI:** 10.3389/fmicb.2016.01846

**Published:** 2016-11-22

**Authors:** Kamelia Osman, Jihan Badr, Khalid S. Al-Maary, Ihab M. I. Moussa, Ashgan M. Hessain, Zeinab M. S. Amin Girah, Usama H. Abo-shama, Ahmed Orabi, Aalaa Saad

**Affiliations:** ^1^Department of Microbiology, Faculty of Veterinary Medicine, Cairo UniversityGiza, Egypt; ^2^Department of Poultry Diseases, Animal Health Research InstituteGiza, Egypt; ^3^Department of Botany and Microbiology, College of Science, King Saud UniversityRiyadh, Saudi Arabia; ^4^Department of Health Science, College of Applied Studies and Community Service, King Saud UniversityRiyadh, Saudi Arabia; ^5^Department of Poultry Diseases, National Research CenterGiza, Egypt; ^6^Department of Microbiology, Faculty of Veterinary Medicine, Sohag UniversitySohag, Egypt

**Keywords:** antimicrobial resistance phenotype, resistance in the quinolone-resistance-determining-regions, biofilm, chicken meat, *mec*A gene

## Abstract

The use of antibiotics in farm management (growing crops and raising animals) has become a major area of concern. Its implications is the consequent emergence of antibiotic resistant bacteria (ARB) and accordingly their access into the human food chain with passage of antibiotic resistance genes (ARG) to the normal human intestinal microbiota and hence to other pathogenic bacteria causative human disease. Therefore, we pursued in this study to unravel the frequency and the quinolone resistance determining region, *mec*A and *cfr* genes of methicillin-susceptible *Staphylococcus aureus* (MSSA), methicillin-resistant *S. aureus* (MRSA), methicillin-resistant coagulase-negative staphylococci (MRCNS) and methicillin-susceptible coagulase-negative staphylococci (MSCNS) isolated from the retail trade of ready-to-eat raw chicken meat samples collected during 1 year and sold across the Great Cairo area. The 50 *Staphylococcus* isolated from retail raw chicken meat were analyzed for their antibiotic resistance phenotypic profile on 12 antibiotics (penicillin, oxacillin, methicillin, ampicillin-sulbactam, erythromycin, tetracycline, clindamycin, gentamicin, ciprofloxacin, chloramphenicol, sulfamethoxazole-trimethoprim, and vancomycin) and their endorsement of the quinolone resistance determining region, *mec*A and *cfr* genes. The isolation results revealed 50 isolates, CPS (14) and CNS (36), representing ten species (*S. aureus, S. hyicus, S. epidermedius, S. lugdunensis, S. haemolyticus, S. hominus, S. schleiferi, S. cohnii, S. intermedius*, and *S. lentus*). Twenty seven isolates were methicillin-resistant. Out of the characterized 50 staphylococcal isolates, three were MRSA but only 2/3 carried the *mec*A gene. The ARG that bestows resistance to quinolones, β-lactams, macrolides, lincosamides, and streptogramin B [MLS(_B_)] in MRSA and MR-CNS were perceived. According to the available literature, the present investigation was a unique endeavor into the identification of the quinolone-resistance-determining-regions, the identification of MRSA and MR-CNS from retail chicken meat in Egypt. In addition, these isolates might indicate the promulgation of methicillin, oxacillin and vancomycin resistance in the community and imply food safety hazards.

## Introduction

Globally, health-conscious consumers have declined from consuming beef red meat which has been linked to heart disease, to choose leaner and easy digestible meats perceiving chicken, a white meat, to be a healthier option (Hathwar et al., 2012). Numerous potential vehicles of transmission of foodborne pathogens exist and commercial chicken meat has been identified as one of the most important food vehicles for these organisms, antimicrobial-resistant bacteria and antimicrobial resistance genes (Phillips et al., 2004; Aarestrup and Schwarz, 2006; Verraes et al., 2013). However, the bearing of foodborne pathogens in poultry meat and its by-products is a disturbing issue in the poultry industry due to its impact on public health and a challenge to the medical and veterinary officials worldwide (APUA, 2010a; Ruban and Fairoze, 2011). Epidemiologically, poultry meat is of paramount importance and still inculpated as prime source of human food poisoning (Kadariya et al., 2014). Although specific data on the burden of foodborne disease associated with *Staphylococcus* in poultry meat has been previously limited (Capita et al., 2002; Pesavento et al., 2007; Persoons et al., 2009), yet it has gained importance in the last couple of years (Bhargava et al., 2011; Hanson et al., 2011; Boost et al., 2013; He et al., 2013; Martins et al., 2013; Momtaz et al., 2013; Yurdakul et al., 2013; Islam et al., 2014; Khallaf et al., 2014; Xin et al., 2014; Abdalrahman et al., 2015; Owuna et al., 2015; Pinto et al., 2015; Bortolaia et al., 2016; Teramoto et al., 2016), it is considered to be significant to be a disturbing issue in the poultry industry due to its impact on public health and a challenge to the medical and veterinary officials worldwide (APUA, 2010a; Ruban and Fairoze, 2011).

The Genus *Staphylococcus* is very well-characterized consisting of 51 species and 27 sub-species (www.bacterio.net/staphylococcus.html). *S. aureus* is the most significant species within this genus, termed as coagulase-positive-*Staphylococcus* (CPS) by virtue of its versatility as a pathogen in humans and animals in addition to its being one of the causes of food intoxication (Jørgensen et al., 2005; Cunha, 2009). Other *Staphylococcus* species, collectively termed coagulase-negative-staphylococci (CNS), have gained importance as they have been implicated to be responsible for a variety of opportunistic infections in humans and animals (Vuong and Otto, 2002), their association with nosocomial infections in neonatal intensive care units and food poisoning in spite of the fact that, they are also not classical food poisoning bacteria (Cortes et al., 2013; Becker et al., 2014; Tong et al., 2015) as they are less pathogenic than *S. aureus* possessing a smaller array of virulence factors (Becker et al., 2014). Due to the ubiquity of many of the species within this group, their clinical significance has traditionally been dismissed, and when isolated from clinical specimens, the bacteria have merely been regarded as contaminants (Becker et al., 2014). This perception is, however, changing as many species have emerged as important causes of nosocomial infections, particularly in relation to foreign device-related infections and infections in immunocompromised patients (Ibrahem et al., 2009; Mathema et al., 2009). Almost half of all the CNS species that have been identified to date have been implicated in human infections (Lowy, 2013) and a PubMed search on CNS results in more than 15,000 references, reflecting the increasing medical impact of these bacteria (Becker et al., 2014).

The propensity for staphylococci to develop antimicrobial resistance is a cause for great concern in both human and veterinary medicine (Vanderhaeghen et al., 2010). Globally, antimicrobial resistance was highlightened as a priority issue (Davies and Davies, 2010; Acar and Moulin, 2012; WHO, 2012; World Economic Forum, 2013; One Health Commission, 2014) due to the increase in the public disquietness about the rampant promulgation of antimicrobial resistant bacteria (AMRB) causing a drop in the food supply as a result of treatment failure of the infected livestock, in addition to curtailment on international animal trade and human traveling. The connection between antibiotic use and antibiotic resistance profiles in chicken and human health has been reviewed by Marshall and Levy (2011) which also included methicillin-resistant *S. aureus* (MRSA) and which has been found in 12% of animal products—beef, veal, lamb, pork, and a variety of fowl—in Denmark (Normanno et al., 2007; de Boer et al., 2009). The European Food Safety Authority, the European Commission, and the European Centre for Disease Prevention, Control (ECDC), the U.S. National Antimicrobial Resistance Monitoring System (NARMS), a collaboration of the Food and Drug Administration (FDA), the Centers for Disease Control and Prevention (CDC), and the U.S. Department of Agriculture, routinely compile surveillance reports on antibiotic resistance in chicken from member countries which includes in some cases methicillin-susceptible *S. aureus* (MRSA). Significant levels of resistance are reported but the patterns vary considerably (CDDEP, 2015). The MRSA associated with animals and its relevance to human health has been highlighted by Pantosti (2012). Although, CNS is recognized as technologically/hygienically very important bacteria in food production and preservation (Hadžiosmanović et al., 2005; Šušković et al., 2010), yet its presence in food is beyond any doubt of public health significance due to the possible promulgation of AMRB and antimicrobial resistant genes (AMRG) (Zdolec et al., 2012a,b, 2013a,b; Dobranić et al., 2013; Chajęcka-Wierzchowska et al., 2015). In previous decades, a continuous loss of susceptibility toward most of the available antibiotics was recorded for CNS with a greater tendency to develop multidrug resistance (Taponen and Pyörälä, 2009) limiting present therapies posing a great threat to the health care system worldwide (Balaban and Rasooly, 2000; Anderson-Berry et al., 2011; deKraker et al., 2011; Jean-Baptiste et al., 2011; Marra et al., 2011). CNS of both animal and human origins are believed to serve as important reservoirs of antimicrobial resistance genes (Becker et al., 2014), which can transfer and integrate into the *S. aureus* genome leading to the emergence of new, potentially more resistant strains (Otto, 2012; Vitali et al., 2014). The mechanisms responsible for antimicrobial resistance in CNS are identical to those occurring in *S. aureus* (Livermore, 2000). Reports of methicillin-resistant strains among CNS are challenging due to the large proportion of methicillin-resistant strains and increasing numbers of isolates reinforcing the need to revise their importance to food safety (Bhargava and Zhang, 2014; Chah et al., 2014; Osman et al., 2016a,b). Therefore, screening of these elements is important for public health and despite the importance of such a screen, limited data are available for CNS at the species level among the chicken retail meat.

Consequently, our endeavor was to reveal the hypothetical implications that chicken meat may serve as a vector for the conveyance of AMRB and AMRG to humans. Therefore, we pursued in this study to probe the quinolone resistance determining region that has not been formerly analyzed, the *mec*A and *cfr* genes of methicillin-susceptible *Staphylococcus aureus* (MSSA), methicillin-resistant *S. aureus* (MRSA), methicillin-resistant coagulase-negative staphylococci (MRCNS) and methicillin-susceptible coagulase-negative staphylococci (MSCNS) isolated from the retail trade of ready-to-eat raw chicken meat sold in the retail market.

## Materials and methods

### Sample collection and processing

Retail chicken (breasts and thighs) products (*n* = 100) were collected from 20 retail supermarkets and groceries in the Great Cairo Area during the year 2013. The purchased products at retail were placed in sterile plastic containment (zip-seal bags) then into ice chests with cold blocks to be immediately sent to the Department of Poultry Diseases, Animal Health Research, Institute, Dokki, laboratory for microbiological analyses. Upon arrival at the laboratory, the samples were transferred aseptically into stomacher bags with sterile tongs and weighed. Samples weighing 205 g were hand agitated with 250 ml of sterile 0.1% peptone broth. A 30 ml aliquot of sample wash was added to 30 ml of Baird Parker broth (2x concentration) with tellurite enrichment in a 250 ml sterile Erlenmeyer flask and incubated at 37°C for 18–24 h. After enrichment, 10 μl of broth was plated onto each of five antibiotic-supplemented Baird Parker agars with EY tellurite enrichment (BD) formulations and one un-supplemented formulation and incubated 18–24 h for growth. After enrichment, 10 μl was plated onto Baird Parker agar (BPA) with EY tellurite enrichment and incubated at 35°C 18–24 h for growth. Ten microliter were also plated onto CHROMagar MRSA plates and incubated at 35°C 24–48 h and examined for growth. Presumptive *S. aureus* (black colonies with clear halos on BPA) and presumptive MRSA (mauve colonies on CHROMagar) were initially screened using conventional methods: Gram staining, coagulase plasma test, hemolysis, catalase production, salt mannitol agar growth, Voges-Proskauer test, the maltose and trehalose fermentation test, susceptibility to polymyxin B test (300 lg, NewProv) and KOH test. Further confirmation was achieved by the API Staph kit. Individual colonies from all positive plates were streaked for isolated colonies twice before being frozen at −80°C in Brucella Broth (BD) containing 20% glycerol (Sigma). On the day of experimentation the isolates were subcultured onto Mueller-Hinton agar, stored at 27°C in tryptic soy broth and fetal calf serum containing 2% yeast extract and revitalized for phenotypic resistance testing and DNA preparation.

### Antibiotic susceptibility test

Antibiotic resistance of the 50 isolated *Staphylococcus* spp. was examined using 12 commercial antibiotic discs and performed by the standard disc diffusion method (CLSI, 2013). *S. aureus* ATCC 25923 was plated as a control. After incubation at 37°C for 24 h the diameter of inhibition halos around the colonies was measured. The antibiotics used were: penicillin (10 μg), ampicillin-sulbactam (20 μg), chloramphenicol (30 μg), ciprofloxacin (5 μg), erythromycin (15 μg), tetracycline (30 μg), gentamicin (10 μg), clindamycin (2 μg), methicillin (5 μg), oxacillin (1 μg), vancomycin (30 μg), and sulfamethoxazole/trimethoprim (25 μg). In the present investigation, we followed the criteria and standardized international terminology for defining multidrug-resistant (MDR), extensively drug-resistant (XDR) and pandrug-resistant (PDR) in *S. aureus* that was created through a joint initiative by the European Centre for Disease Prevention and Control (ECDC) and the Centers for Disease Control and Prevention (CDC) (Magiorakos et al., 2012): MDR was defined as acquired non-susceptibility to at least one agent in three or more antimicrobial categories (and one or more of these have to apply: an MRSA is always considered MDR by virtue of being an MRSA or/and non-susceptible to ≥1 agent in ≥3 antimicrobial categories), XDR was defined as non-susceptibility to at least one agent in all but two or fewer antimicrobial categories (non-susceptible to ≥1 agent in all but ≤2 categories) and PDR was defined as non-susceptibility to all agents in all antimicrobial categories.

### Extraction of staphylococcal DNA and amplification of the 16*S* rRNA gene, quinolone resistance determining region, *mec*A and *cfr* genes by PCR

Bacterial genomic DNA was made from all 50 isolates from 2 mL of bacterial suspension from an overnight culture in brain-heart infusion using a protocol previously described (Bakshi et al., 2005). The DNA pelletts were resuspended in an appropriate volume of TE solution (10 mM Tris-HCl, pH 8.0; 1 mM EDTA). Amplification was performed from the purified genomic DNA and a 0.2 mM concentration of each of the *Staphylococcus*-genus-specific primers (16*S* rRNA gene) was used. An internal control was integrated into the PCR-based assays to verify the efficiency of the amplifications and to ensure that significant PCR inhibition was absent. Genus-specific confirmation was carried out by PCR using the primers: F: AAC TCT GTT ATT AGG GAA GAA CA and R: CCA CCT TCC TCC GGT TTG TCA CC with annealing temperature: 68°C for 45 s to reveal an amplicon of bp 756 (Zhang et al., 2004). *S. aureus* ATCC 43300 was used as the positive control, while *E*. *coli* NCIMB 50034 was used as the negative control.

The confirmed staphylococci isolates were confirmed as MRSA using PCR to detect the presence of *mec*A (the gold-standard reference method for this analysis). Four additional antimicrobial resistance genes frequently reported in *S. aureus* conferring resistance in the quinolone-resistance-determining-regions (QRDRs) responsible for quinolone resistance (*gyrA, gyr*B, and *grlA* genes) and the *cfr* gene conferring resistance to several classes of antibiotics (oxazolidinones, phenicols, streptogramin compounds, lincosamidins, and pleuromutilins known as the PhLOPSA phenotype) were also amplified. Gene's targets, primers pair, nucleotide sequences, operative protocols, PCR amplification was performed as described previously (Table 1). Positive and negative controls were included with all PCR runs performed. All PCR assay runs incorporated a reagent control (without template DNA), positive and negative control (*Streptococcus pyogenes* ATCC 19615) processed in a fashion similar to that of the tested isolates. Three independent PCR amplifications were carried out with a GeneAmp PCR System 2400 (Perkin-Elmer, Weiterstadt, Germany). Two microliters of template DNA was added to 23 μL of distilled sterile water, and finally 25 μL of reaction mixture containing 10 mM Tris-HCl (pH8.3), 50 mM KCl, 2.5 mM MgCl_2_, 100 mM deoxynucleoside triphosphates, 1 mM of each primer and 3U of Taq polymerase were added. The amplicons were resolved by electrophoresis on 1.5% (w/v) agarose gel in TBE buffer, stained with ethidium bromide and photographed using a Polaroid Camera.

**Table 1:** **Antimicrobial resistance genes targets, primers, and nucleotide sequences**.

**Gene designation**	**Oligonucleotide sequences (5′-3′)**	**Amplion size (bp)**	**PCR conditions**	**References**
*mecA*	F: GTAGAAATGACTGAACGTCCGATAA	310	Initial denaturation: 95°C for 1 min	Spanu et al., 2004
	R: CCAATTCCACATTGTTTCGGTCTAA		Amplification (30 cycles of)	
			Denaturation: 95°C for 30 s	
			Annealing: 50°C for 30 s	
			Extension: 72°C for 60 s	
			Final extension: 72°C for 4 min	
*cfr*	F: TGAAGTATAAAGCAGGTTGGGAGTCA	746	Initial denaturation: 94°C for 1 min	Kehrenberg and Schwarz, 2006
	R: ACCATATAATTGACCACAAGCAGC		Amplification (34 cycles of)	
			Denaturation: 94°C for 1 min	
			Annealing: 48°C for 30 s	
			Extension: 72°C for 3 min	
			Final extension: 72°C for 7 min	
*gyrA*	F: ATGGCTGAATTACCTCAATC	399	Initial denaturation: 95°C for 1 min	
	R: CATCATAGTTATCGATGAAATC		Amplification (30 cycles of)	Dubin et al., 1999
			Denaturation: 95°C for 30 s	
			Annealing: 55°C for 30 s	
*grlA*	F: AATACGTATGATAAGAATTTCCG	429	Extension: 72°C for 60 s	
	R: GTTGTGTCATCATAGTTTGG		Final extension: 72°C for 4 min	
*gyrB*	F: CAGCGTTAGATGTAGCAAGC	250		Linde et al., 2001
	R: CCGATTCCTGTACCAAATGC			

## Results

The prevalence and diversity of the *Staphylococcus* species isolated from the chicken meat were found to be: out of the 100 chicken meat samples, 50 *Staphylococcus* species were isolated, which were further identified as, 14/50 CPS isolates differentiated into, *S. aureus* (*n* = 3), *S. hyicus* (*n* = 10), *S. intermedius* (*n* = 1); and 36/50 CNS isolates identified as *S. epidermidis* (*n* = 13), *S. lugdunensis* (*n* = 15), *S. hemolyticus* (*n* = 1), *S. hominus* (*n* = 3), *S. lentus* (*n* = 1), *S. schleiferi* (*n* = 2), and *S. cohnii* (*n* = 1).

### Resistance phenotypes of the *Staphylococcus* isolates

Table 2 shows the resistance phenotype of each of the 50 *Staphylococcus* isolates. Complete resistance to penicillin, gentamycin, clindamycin, oxacillin, and sulfamethoxazole/trimethoprim, with resistance to other important antimicrobials was also observed. The antimicrobial resistance profile of the 50 isolated *Staphylococcus* spp to different antibiotics was analyzed; none of the isolates were totally sensitive to the 12 tested antibiotics and 16/50 of the isolates were shown to be resistant to at least 3 antibiotics (*S. lugdunensis*) representing three classes. A small percentage of the isolates (less than 50%) demonstrated resistance to oxacillin, methicillin, erythromycin, chloramphenicol, ciprofloxacin, vancomycine, and tetracycline (Table 2). Also resistant to β-lactams, such as ampicillin (6/50), penicillin (22/50), methicillin (8/50), and oxacillin (13/50) was evident (Table 2). Forty seven out of the fifty isolates were resistant to penicillin and tetracycline.

**Table 2:** **Distribution of ***Staphylococcus*** spp. isolated from chicken meat samples according to their species diversity and multidrug resistance pattern**.

**Coagulase reaction**	***Staphylococcus* spp. (*n* = isolates)**	**Antibiotics**											
		**Ampicillin-sulbactam**	**Chloramphenicol**	**Ciprofloxacin**	**Clindamycin**	**Erythromycin**	**Gentamycin**	**Methicillin**	**Oxacillin**	**Peniciilin**	**Sulfamethoxazole/Trimethoprim**	**Tetracycline**	**Vancomycin**
**Resistance Pattern**													
Coagulase positive	*S. aureus* (3)	1	1	0	3	3	3	3	3	3	3	3	2
	*S. hyicus* (10)	4	6	5	9	9	6	6	8	9	9	9	7
	*S. intermedius* (1)	1	1	1	1	1	1	0	1	1	1	1	1
Coagulase negative	*S. epidermidis* (13)	3	1	5	13	11	10	7	9	12	4	11	3
	*S. lugdunensis* (15)	1	1	3	15	14	12	6	8	14	9	15	4
	*S. hemolyticus* (1)	1	1	0	1	1	1	1	1	1	1	1	1
	*S. hominus* (3)	0	2	0	3	3	3	2	3	3	2	3	1
	*S. lentus* (1)	1	1	1	1	1	1	1	1	1	1	1	1
	*S. schleiferi* (2)	0	0	1	2	2	2	2	2	2	2	2	0
	*S. cohnii* (1)	0	0	0	1	1	1	0	1	1	1	1	0
Total	50	12	14	16	49	46	40	27	37	47	33	47	20

### The distribution of antibiotic resistance ratios of the *Staphylococcus* (S) Coagulase positive (CP)-negative (CN) that are resistant (MR) and susceptible to methicillin (MS)

Methicillin resistance in CPS and CNS was calculated to be 64.3 and 52.8%, respectively. The antimicrobial resistance patterns of the isolates are shown in Table 3. The resistance ratios to clindamycin, methicillin, oxacillin, penicillin, and sulfamethoxazole/trimethoprim in CPS and to clindamycin, gentamycin, methicillin, oxacillin and penicillin in CNS were found to be higher in the methicillin-resistant isolates compared to those that were susceptible to methicillin (*p* < 0.001). The lowest resistance ratio in the methicillin—resistant staphylococci was detected for ciprofloxacin and ampicillin-sulbactam in the CPS and to ampicillin-sulbactam, chloramphenicol and ciprofloxacin in the CNS. A comparison of the resistance patterns revealed that all isolates from chickens showed resistance from three to nine classes of antimicrobial agents (Table 4).

**Table 3:** **The antibiotic resistance ratios of the ***Staphylococcus*** (S) Coagulase positive (CP)– negative (CN) that are resistant (MR) and susceptible to methicilin (MS) (%)**.

**Number of staphylococci = 50**	**CPS** ***n*** = 14.50 **(28%)**		**CNS** ***n*** = 36.50 **(72%)**	
**Antibiotics**	**MRCPS (9/14, 64.3%)**	**MSCPS (5/14, 35.7%)**	**MRCNS (19/36, 52.8%)**	**MSCNS (17/36, 47.2%)**
Ampicillin-sulbactam	28.6	14.3	16.7	0
Chloramphenicol	35.7	21.4	11.1	5.6
Ciprofloxacin	21.4	21.4	19.4	8.3
Clindamycin	57.1	35.7	52.8	47.2
Erythromycin	42.9	50.0	50.0	41.7
Gentamycin	42.9	28.6	52.8	30.6
Methicillin	64.3	35.7	52.8	47.2
Oxacillin	64.3	21.4	52.8	16.7
Peniciilin	64.3	28.6	52.8	41.7
Sulfamethoxazole/Trimethoprim	64.3	28.6	38.9	16.7
Tetracycline	57.1	35.7	47.2	47.2
Vancomycin	50.0	21.4	27.8	0

**Table 4:** **Multidrug resistance profiles and resistance genes of CPS and CNS from chicken meat**.

***Staphylococcus* spp**.	**Phenoptypic resistance combination pattern**	***n* = antibiotics**	***n* = classes**	**Resistance genes**				
				***mecA***	***gyrA***	***grlA***	***gyrB***	***cfr***
**COAGULASE POSITIVE**								
*S. aureus*	P, OX, MET, CN, E, DA, TET, VAN, SXT	9	7	ND	+	+	ND	ND
	P, OX, MET, SAM, CN, E, DA, TET, SXT	9	6	+	ND	ND	+	ND
	P, OX, MET, CN, E, DA, C, TET, VAN, SXT	10	8	+	+	+	ND	ND
*S. hyicus*	E, CN, TE, VA, SXT	5	5	ND	ND	ND	+	ND
	P, OX, MET, SAM, SXT	5	2	ND	ND	ND	ND	ND
	P, CN, E, DA, C, TE, VA	7	7	ND	ND	ND	+	ND
	P, OX, CN, E, DA, TET, SXT, CIP	8	7	ND	+	+	ND	ND
	P, OX, ME, CN, E, DA, TE, VA, SXT	9	7	ND	+	+	ND	ND
	P, OX, MET, SAM, E, DA, C, TE,VA, SXT	10	7	+	ND	+	ND	ND
	P, OX, ME, CN, E, DA, C, TE, VA, SXT, CIP	11	10	+	+	+	ND	ND
	P, OX, MET, SAM, E, DA, C, TE,VA, SXT, CIP	11	8	+	+	+	ND	ND
	P, OX, MET, CN, E, DA, C, TET, VAN, SXT, CIP	11	9	+	+	+	+	ND
	P, OX, SAM, CN, E, DA, C, TET, VAN, SXT, CIP	11	9	ND	+	+	+	ND
*S. intermedius*	P, OX, SAM, CN, E, DA, C, TE,VA, SXT, CIP	11	9	ND	ND	+	+	ND
**COAGULASE NEGATIVE**								
*S. epidermidis*	P, E, DA, TET	4	4	ND	ND	ND	ND	ND
	P, E, CN, SXT	4	4	ND	ND	ND	ND	ND
	P, E, DA, TET	4	4	ND	ND	ND	ND	ND
	CN, E, DA, TET	4	4	ND	ND	ND	ND	ND
	P, Ox, CN, DA, TET, CIP	6	5	ND	+	+	ND	ND
	P, OX, MET, CN, DA, SXT	6	4	ND	ND	ND	+	ND
	P, OX, CN, E, DA, TET, CIP	7	6	ND	+	+	ND	ND
	P, OX, MET, CN, E, DA, TET	7	5	ND	+	+	ND	ND
	P, OX, MET, SAM, CN, E, DA, TET	8	5	+	+	ND	ND	ND
	P, OX, MET, SAM, CN, E, DA, TET, CIP	9	6	+	+	+	+	ND
	P, OX, MET, SAM, CN, E, DA, TET, VAN	9	6	+	ND	ND	+	ND
	P, OX, MET, CN, E, DA, TET, VAN, SXT,CIP	10	8	ND	+	+	+	ND
	P, OX, MET, CN, E, DA, C, TET, VAN, SXT, CIP	11	9	+	+	+	+	+
*S. lugdunensis*	E, DA, TET	3	3	ND	+	ND	ND	ND
	P, CN, E, DA, TET	5	5	ND	ND	ND	ND	ND
	P, CN, E, DA, TET	5	5	ND	+	+	+	ND
	P, E, DA, C, TET, SXT	6	6	ND	ND	+	ND	ND
	P, CN, E, DA, TET, CIP	6	6	ND	+	+	ND	ND
	P, OX, CN, DA, TET, CIP	6	5	+	+	+	ND	ND
	P, E, DA, C, TE, VA, SXT	7	7	ND	+	ND	ND	ND
	P, OX, MET, CN, E, DA, TET	7	5	ND	ND	ND	ND	ND
	P, CN, E, DA, TET, VAN, SXT	7	7	ND	+	ND	+	ND
	P, OX, MET, CN, E, DA,TE, VA, SXT	9	7	ND	ND	+	ND	ND
	P, OX, MET, CN, E, DA, TET, SXT, CIP	9	7	ND	+	+	ND	ND
	P, OX, MET, CN, E, DA, TET, VAN, SXT	9	7	+	ND	ND	+	ND
	P, OX, MET, CN, E, DA, TET, VAN, SXT	9	7	+	+	+	+	ND
	P, OX, MET, CN, E, DA, TET, VAN, SXT	9	7	ND	+	+	ND	ND
	P, OX, MET, SAM, CN, E, DA, TET, SXT	9	6	+	ND	+	ND	ND
*S. hemolyticus*	P, OX, MET, SAM, CN, E, DA, C, TET, VAN, SXT	11	8	+	+	+	+	+
*S. hominus*	P, OX, CN, E, DA, C, TET	7	6	ND	+	ND	ND	ND
	P, OX, MET, CN, E, DA, TET, SXT	8	6	+	ND	+	+	ND
	P, OX, MET, CN, E, DA, C, TET, VAN, SXT	10	8	+	+	ND	+	ND
*S. schleiferi*	P, OX, MET, CN, E, DA, TET, SXT	8	6	ND	ND	ND	+	ND
	P, OX, MET, CN, E, DA, TET, SXT, CIP	9	7	+	+	+	ND	ND
*S. cohnii*	P, OX, CN, E, DA,TET, SXT	7	6	ND	+	ND	+	ND
*S. lentus*	P, OX, MET, SAM, CN, E, DA, C, TE,VA, SXT, CIP	12	9	+	+	+	ND	+

*C, Chloramphenicol; CIP, Ciprofloxacin; CN, Gentamycin; DA, Clindamycin; E, Erythromycin; MET, Methicillin; OX, Oxacillin; P, Penicillin; SAM, Ampicillin-sulbactam; SXT, Sulfamethoxazole/Trimethoprim; TE, Tetracycline; VA, Vancomycin; ND, not detected*.

Eight isolates showed resistance to oxacillin, undetectable by *mecA* amplification. The three *S. aureus* isolates were resistant to 9–10 antimicrobials (Table 4). Two unique susceptibility profiles were identified among the *S. aureus* isolates, with many resistant to multiple clinically important antimicrobial classes (Table 4). In addition, it was noticed that 49/50 *Staphylococcus* isolates were MDR (non-susceptible to ≥1 agent in ≥3 antimicrobial classes) while 1/10 of the *S. hyicus* did not apply to this as it was only resistant to two classes of antibiotics although the number of antibiotics were five (P, OX, MET, SAM, and SXT). *S. lentus* was the only species to be PDR as it was resistant to the 12 antibiotics (P, OX, MET, SAM, CN, E, DA, C, TE, VA, SXT, and CIP) which represented nine classes and consequently, the remaining 48 *Staphylococcus* isolates were XDR as they were resistant to ≥1 agent in all but ≤2 classes (Table 4).

### Antimicrobial resistance genes

Of the 50 *Staphylococcus* isolates, two of the CPS species (2/3 *S. aureus*; 4/10 *S. hyicus*) were identified positive for *mecA* (6/50) while six out of the seven CNS species expressed the *mec*A gene (4/13 *S. epidermedius*; *S. lugdunensis* 4/15; *S. haemolyticus* 1/1; *S. hominus* 2/3; *S. schleiferi* 1/2 and *S. lentus* 1/1) (Table 4). With the exception of 1/8 *S. lugdunensis* isolates which was susceptible to oxacillin, *mecA*-positive, four *Staphylococcus* isolates (3/10 *S. hyicus*; 1/1 *S. intermedius*) were resistant to ciprofloxacin, gentamicin, erythromycin, methicillin, oxacillin, penicillin, sulfamethoxazole/trimethoprim, and vancomycin (Table 4). Forty seven out of the fifty isolates (47/50) were resistant to penicillin and tetracycline, while only one was resistant to chloramphenicol. Methicillin-resistant non-*Staphylococcus aureus* (MRNSA) isolates were resistant to nine of the 12 antimicrobials on the panel, including one to gentamicin.

Interestingly, two observations were recorded: (1) one of the three *S. aureus* did not carry the *mecA* gene and was not even phenotypically characterized as MRS (Table 5); and (2) the resistance gene *mecA* was present in the *S. lugdunensis* isolate (1/8) and which was phenotypically characterized as methicillin-susceptible non-*Staphylococcus aureus* (MSNSA) but at the same time phenotypically oxacillin resistant (Table 4). The resistance gene tested *mecA* was detected in 16/26 methicillin-resistant non-*Staphylococcus aureus* (MRNSA) isolates (Table 5), while *cfr* was not detected in any of the isolates (Table 4). Of the three MSSA isolates, two carried the *gyrA* gene and one carried the *gyrB* gene (Table 4). The 50 isolates of *Staphylococcus* species namely: *aureus, hyicus, simulans, lugdunensis, hominus, hemolyticus, epidermidis, intermedius*, and *S. sciuri* were non-*cfr*-carriers. Our susceptibility testing showed that, all non-*cfr*-carrying staphylococci (23/50) were resistant to the 12 antibiotics tested in the present with varying degrees. Of the 50 isolates, 40/50 isolates were gentamicin-resistant, 37/50 were oxacillin resistant while only 16/50 were ciprofloxacin-resistant. The results of antimicrobial susceptibility testing indicated that the non-*cfr*-carrying CNS exhibited also expanded resistance to antimicrobial agents other than those included in the *cfr*-mediated PhLOPSA phenotype (Table 4) [The *Cfr* rRNA Methyltransferase confers resistance to several classes of antibiotics (phenicols, lincosamides, oxazolidinones, pleuromutilins, and streptogramin A; PhLOPSA phenotype)]. It was possible to conclude that 25/34 of the MR-NSA strains were potentially methicillin resistant. However, these results were evidently different from those obtained by PCR, in which the gene *mec*A was detected in only 16/47 of the CNSA isolates (Table 5).

**Table 5:** **Prevalence of ***mecA*** gene in chicken meat samples**.

***Staphylococcus* spp. (*n* = isolates)**	***n* = of MRS**	**Precence of *mecA* gene**		***n* = of MSS**	**Precence of *mecA* gene**	
		***n =***	**%**		***n =***	**%**
**COAGULASE POSITIVE**						
*S. aureus* (*n* = 3)	3	2	66.7	0	0	0
*S. hyicus* (*n* = 10)	6	4	66.7	4	0	0
*S. intermedius* (*n* = 1)	0	0	0	1	0	0
**COAGULASE NEGATIVE**						
*S. epidermedius* (*n* = 13)	7	4	57.1	6	0	0
*S. lugdunensis* (*n* = 15)	7	3	0	8	1	12.5
*S. haemolyticus* (*n* = 1)	1	1	100	0	0	0
*S. hominus* (*n* = 3)	2	2	100	1	0	0
*S. schleiferi* (*n* = 2)	2	1	50	0	0	0
*S. cohnii* (*n* = 1)	0	0	0	1	0	0
*S. lentus* (*n* = 1)	1	1	100	0	0	0
Total (*n* = 50)	29 (58%)	18	62.1	21 (42%)	1	3.5

## Discussion

In previous investigations, the frequency of *S. aureus* in the chicken meat was found to be 33.3% in The Netherlands, 4% in Egypt, 22.7% in Switzerland and 65% in Japan (Schraft et al., 1992; Bakr et al., 2004; Kitai et al., 2005). The authors traced back the source of contamination to particular slaughterhouses in Switzerland while in Egypt poor hygienic and sanitary conditions in addition to the contaminated butchers and meat handlers during the process of slaughtering and evisceration of the bird (Kluytmans et al., 1995; Kitai et al., 2005) could be the main cause for the 50% prevalence recorded in the present investigation.

The coexistence of two subpopulations (one susceptible and the other resistant) within a culture of staphylococci could hinder an explicit detection of oxacillin/methicillin resistance (Bannerman, 2003). All cells in a culture may carry the genetic information for resistance, but only a small number may express the resistance *in vitro*. This phenomenon is termed heteroresistance and occurs in staphylococci resistant to penicillinase-stable penicillins, such as oxacillin. Cells expressing heteroresistance grow more slowly than the oxacillin-susceptible population and may be missed at temperatures above 35°C. This is why CLSI recommends incubating isolates being tested against oxacillin for a full 24 h before reading (CLSI, 2013). Oxacillin maintains its activity during storage better than methicillin and is more likely to detect heteroresistant strains. Previously, Petrelli et al. (2008) found a significant correlation between oxacillin resistance and resistance to erythromycin, clindamycin, gentamycin and ciprofloxacin, which is in queue with our findings; but on evaluating our results, penicillin and tetracycline resistance were also found to be significantly correlated with oxacillin resistance. It should be noted that, widespread veterinary usage of the fluoroquinolones, particularly in Europe, has been implicated (CVM, 2009; CVM/FDA, 2015) and that being a second-generation fluoroquinolone, is on the World Health Organization's List of Essential Medicines and the most important medications needed in a basic health system (WHO, 2015). Fearfully, this could lead to a rapid and sudden evolvement of resistance to ciprofloxacin and other fluoroquinolones, even during a course of treatment. This documentation is consistent with antecedent investigations and substantiates the allegations of MRSA causing healthcare-associated infections as being MDR to the traditionally prescribed drugs (erythromycin, clindamycin, fluoroquinolones, and tetracycline), while strains inducing community-associated infections are generally solely resistant to ß-lactam agents, erythromycin while feasibly fluoroquinolone resistant (Oliveira and de Lencastre, 2002).

Since 1996, MRSA strains with decreased susceptibility to vancomycin and strains fully resistant to vancomycin have been reported (CDC, 2013) a fact which should be taken into consideration in the Egyptian community as we detected a 20% resistance to vancomycin. Methicillin resistant *Staphylococcus aureus* (MRSA) and vancomycin resistant *Staphylococcus aureus* (VRSA) are some of the prominent pathogens that cause a wide variety of infections in humans and animals (Venkatesh et al., 2006; van Loo et al., 2007; Plata et al., 2009; Cuny et al., 2010; Gould, 2010; Grundmann et al., 2010). It should be emphasized that the present investigation highlights the importance of a sustainable surveillance program for any signs of vancomycin resistance emergence in Egypt concerning the medical and veterinary health condition in conjunction to typing and origin of the isolates. An increase in the cases of incompetent antibiotic treatment and mortality rates in avian disease (Han et al., 2013) is a common outcome of methicillin resistance, generating critical community health concern. Considering the limited options disease medication caused by MDR staphylococcal species, it is worrisome that CNS resistant to broad-spectrum antibiotics have been introduced in the community through the food chain. Regarding the CNS identified in this study, *S. cohnii* and *S. lentus* have been signified to yield enterotoxins (Podkowik et al., 2013).

Batista et al. (2013) have shown that oxacillin is more sensitive for predicting methicillin resistance, since 100% of isolates containing the *mec*A gene were phenotypically resistant to oxacillin, whereas only 13.3% were resistant to cefoxitin. Thus, the test using cefoxitin may not accurately reflect the phenotypic resistance of CNS, which has also been observed by other authors (Frigatto et al., 2005; Hung et al., 2011). To avoid misinterpretation, the detection of the *mec*A gene (the commonest gene that imparts oxacillin resistance in staphylococci) by nucleic acid amplification tests, such as the PCR, is currently considered the criterion standard for identification of MRSA strains (Zhang et al., 2004, 2008; Bagcigil et al., 2007; Zaraket et al., 2007), but this has not yet been established for CNS. The risk of transmission of resistance genes by horizontal interspecies transfer was clear and has been described by others (Bloemendaal et al., 2010; von Wintersdorff et al., 2016).

Foodborne pathogens, such as MRSA have become a global problem after being in North America, Europe and Asia. MRSA in chicken meat showed its highest prevalence from Germany (37.2%) (Feßler et al., 2011) followed by Netherlands (16.0%) (de Boer et al., 2009). On the other hand, low levels of MRSA in chicken meat were recorded in Japan (Kitai et al., 2005), Jordan (Quddoumi et al., 2006) to reach 0.3% in Korea (Lim et al., 2010) and 0% in Austrai (Zarfel et al., 2014). There are also reports of now the fourth phase has also been started which shows evidences that both animal and human MRSA has been detected on meat. Studies in Japan and Korea reported human MRSA from chicken meat (Lee, 2003; Kitai et al., 2005). Taiwanese study reported MRSA in chicken carcasses (Lin et al., 2009). MRSA have also been detected from the foods, such as meat products and raw chicken meat (Vanderhaeghen et al., 2010). Regardless of the ongoing endorsed methodizes for the MRSA susceptibility testing, Lee et al. (2004) demonstrated a variance in the phenotypic expression of resistance being affected by the cellular growth conditions and that several MRSA isolates which were phenotypically resistant to oxacillin, did not encode the *mec*A gene. MRSA can be found worldwide and Doyle et al. (2011) summarized the global survival results in raw meats, which indicated that the incidence of MRSA was infrequent in poultry meat when compared to the beef and pork meats and with variable prevalences between regions.

Antibiotic resistance patterns of individual pathogens to the drugs used to treat them vary considerably between and within countries. The discrepancies and uncomparable results between researchers, including us, was incriminated by Kluytmans et al. (1995), Chaibenjawong and Foster (2011), Doyle et al. (2011) and Omurtag et al. (2013) to be due to several criteria, such as: (i) sampling and culture methods differed among the studies; (ii) the condition of the meat when being sold is it packed or un-packed in the supermarkets or through the widely spread butcher shops in Great Cairo; (iii) MRSA carriers can contaminate meat during slaughter and processing; (iv) pathogen load on raw meat changes according to species; (v) heat treatment and cross-contamination change the pathogen titre on the meat; (vi) serving frequency and size, and demographic data; (vii) regional differences and social groups dissimilarities are conditions that must be taken into consideration; (viii) infected/colonized food handlers are favorable causes for meat contamination (ix) under conditions of temperature abuse, MSSA and MRSA cells could multiply on meat; (ix) the unjuridical use of antibiotics for animals; (x) different patterns of antibiotic use; (xi) distinct national disease burdens; (xii) disparities in access to first—and second-line treatments; and (xiii) the burden of coinfections.

Eventually, AMR drugs have become a global public health crisis implicating the importance of preventing contamination of food with MRSA, which could have trade consequences and exert coercion to those sovereignties with un-controlled antimicrobial practices to create and enforce proper danger administration strategies. Therefore, the use of antibiotics for animals and their documentation has become a must to aid in analyzing their use and consequent impact on public health to enact policy changes on the consumption of antibiotics (APUA, 2010b; WHO, 2013).

## Conclusion

The occurrence of CNS in food should not be ignored nor their pathogenic potential considered as insignificant as a food-borne pathogen, rather safety measures should be taken to reduce or totally eliminate their occurrence in foods. The incidence of CNS may render food unsafe, as the clinical isolates have been reported to exude virulent traits (Fowoyo and Ogunbanwo, 2016). Our findings emphasize the need for improved hygiene practices during food processing and also during the distribution and consumption of the final food products to avoid the presence of CNS and CPS isolates in meat. The present study cynosures that the MRS has entered the food chain in Egypt through the raw chicken meat constituting a health hazard to consumers and should be given importance due to their zoonotic importance. This necessitates the urgent implementation of an energetic policy to ensure microbiological food safety to prevent the spread of MRS through the food chain by contamination of raw meat. In addition, these isolates might indicate the promulgation of methicillin, oxacillin, and vancomycin resistance in the community and imply food safety hazards.

## Author contributions

KO conceived, designed the experiments, analyzed the data and wrote the paper; KA, IM, AH, ZAG, and UA contributed their scientific advice during the work and MS revision; JB, AO, and AA performed the experiments, supplied and contributed reagents/materials/analysis tools.

### Conflict of interest statement

The authors declare that the research was conducted in the absence of any commercial or financial relationships that could be construed as a potential conflict of interest.
